# Self-reported (IFIS) versus measured physical fitness, and their associations to cardiometabolic risk factors in early pregnancy

**DOI:** 10.1038/s41598-021-02149-7

**Published:** 2021-11-22

**Authors:** Maria Henström, Marja H. Leppänen, Pontus Henriksson, Emmie Söderström, Johanna Sandborg, Francisco B. Ortega, Marie Löf

**Affiliations:** 1 Department of Biosciences and Nutrition, Karolinska Institutet, 141 83 Huddinge, Sweden; 2 Division of Society and Health, Department of Health, Medicine and Caring Sciences, Linköping University, 581 83 Linköping, Sweden; 3 Folkhälsan Research Center, Helsinki, Finland; 4 Faculty of Medicine, University of Helsinki, Helsinki, Finland; 5 PROFITH (PROmoting FITness and Health through Physical Activity) Research Group, Department of Physical Education and Sports, Faculty of Sport Sciences, Research Institute of Sport and Health, University of Granada, Granada, Spain

**Keywords:** Risk factors, Lifestyle modification

## Abstract

Physical fitness is a strong marker of health, but objective fitness measurements are not always feasible. The International FItness Scale (IFIS) for self-reported fitness is a simple-to-use tool with demonstrated validity and reliability; however, validation in pregnancy needs to be confirmed. Also, its association with cardiometabolic health in pregnant women is unknown. Hence, we examined (1) the validity of the IFIS with objectively measured fitness, and (2) the associations of self-reported versus objectively measured cardiorespiratory fitness (CRF) and muscular strength with cardiometabolic risk factors in early pregnancy. Women (n = 303) from the HealthyMoms trial were measured at gestational week 14 for: CRF (6-min walk test); upper-body muscular strength (handgrip strength test); self-reported fitness (IFIS), body composition (air-displacement plethysmography); blood pressure and metabolic parameters (lipids, glucose, insulin). Higher self-reported fitness was associated with better measured fitness (ANOVA overall p < 0.01 for all fitness types), indicating the usefulness of the IFIS in pregnancy. Furthermore, higher self-reported overall fitness and CRF were associated with lower cardiometabolic risk scores (ANOVA p < 0.001), with similar results shown for measured CRF (ANOVA p < 0.001). The findings suggest that IFIS could be useful to stratify pregnant women in appropriate fitness levels on a population-based level where objective measurement is not possible.

## Introduction

### Background

Physical fitness is a powerful marker of health. Cardiorespiratory fitness (CRF) has been considered as an indicator of cardiometabolic health status in both children and adults^1,2^, and greater muscular strength in adults has also been related to better health^3^. In pregnant women, improved physical fitness has been associated with better neonatal outcomes and decreased risk of caesarean section^4^ as well as with less bodily pain and reduced pain disability^5^. Therefore, accurate and feasible measures of physical fitness during pregnancy is essential in epidemiological and public health research. However, since pregnancy is characterized by weight gain as well as other physiological and biochemical changes^6^, this may hinder objective measurement of physical fitness. Thus, there is a need for alternative methods that can accurately assess physical fitness in cases where objective measuring is not possible.

The International FItness Scale (IFIS) has been proposed as a reliable and valid tool in assessing physical fitness levels in a time- and cost-effective way^7^. So far, the IFIS has been validated in several study populations of different ages in Europe^7–11^ and South America^12,13^; yet, only one of these studies has investigated the validity in pregnant women (n = 106)^9^. That study reported that IFIS might be a potential tool in identifying physical fitness level during pregnancy, and their results especially indicate its usefulness to identify women with low physical fitness, which is the group with markedly higher risk of poorer health outcomes^9^. Therefore, the validity of the IFIS in pregnant women should be confirmed in other populations in order to expand its generalizability and incorporate this complementary tool in clinical practice and large-scale studies.

Cardiovascular diseases cause remarkable individual, public health, and economic burden globally^14,15^. Moreover, cardiovascular health during pregnancy is not only linked to pregnancy-related outcomes but is also predictive of latent cardiovascular diseases and future health of the women^16^. Also, obesity during pregnancy has been found to be a major risk factor for adverse outcomes for both mother and fetus/child^17^ although the traditional measure, body mass index (BMI), may not accurately evaluate body fatness in pregnancy^18^. Since women go through regular medical screening during pregnancy, pregnancy has been proposed as a unique opportunity to identify women with higher cardiometabolic risk factors^16,19,20^. A previous study in young adults showed that self-reported CRF as assessed by IFIS predicted cardiometabolic risk factors nearly as well as objectively measured CRF^8^. However, the associations of self-reported physical fitness with cardiometabolic risk factors in pregnant women is unknown, since the only previous studies using IFIS in pregnant women focused on other health outcomes (i.e., health-related quality of life, pain, poor sleep and tiredness-fatigue during pregnancy)^5,9,21^. Thus, in the present study, we aimed to (1) examine the validity of the IFIS for self-reported physical fitness with objectively measured fitness (assessed by a 6-min walk test and handgrip test) as criterion, and (2) investigate the association of self-reported (IFIS) versus objectively measured CRF and muscular strength with body composition and cardiometabolic risk factors in early pregnancy.

## Methods

### Study design, data collection and participants

The present study is a cross-sectional analysis utilizing data from an mHealth intervention in pregnant women: the HealthyMoms trial (clinicaltrials.gov; NCT03298555). A full description of the HealthyMoms study design and methodology has been provided elsewhere^22,23^. In brief, this study was a 2-armed parallel randomized controlled trial aimed at investigating whether a smartphone app (the HealthyMoms app) could promote healthy dietary and physical activity behaviors, and support a healthy weight gain during pregnancy. A total of 305 women were enrolled in the 6-months trial between October 2017 and March 2020 in the county of Östergötland, south of Sweden, and the main results have been published elsewhere^24^. The women had a single pregnancy (i.e., no twins or triplets) and had no prior diagnosis of diabetes type 2 or cardiovascular disease.

For the purpose of the present study, data from the baseline measurements were utilized. These measurements were conducted in early pregnancy (13.9 ± 0.7 gestational weeks) and took place prior to randomization and access to any intervention content (i.e., the HealthyMoms app). At the baseline measurement at Linköping University hospital, Sweden, all enrolled participants provided a fasting blood sample, had their body composition measured, performed physical fitness tests, and completed questionnaires. The questionnaires included background information such as questions regarding age, country of birth, pre-pregnancy weight, parity, occupation and educational attainment, but also questions to assess self-reported fitness. Participants were included in the present study if they had valid data on both of the measured fitness tests, i.e., 6-min walk test to assess CRF and handgrip strength test to assess muscular strength. Due to pelvic girdle pain and recent pneumonia, two individuals were not able to perform the 6-min walk test. Thus, data on a total of 303 women were included in the analyses.

### Ethics

The HealthyMoms trial has been approved by the Regional Ethical Review Board in Linköping, Sweden (DNR: 2017/112-31 and 2018/262-32) and written informed consent was provided by all participating women before entering the trial. The study was conducted in accordance with the World Medical Association Declaration of Helsinki.

### Self-reported physical fitness

Self-reported fitness was assessed through the IFIS scale, which was originally developed to be used in adolescents within the project HELENA (Healthy Lifestyle in Europe by Nutrition in Adolescence), as a tool to assess physical fitness in larger study populations^7^. The IFIS consists of a Likert-type scale ranging from 1–5, where higher IFIS score indicates greater self-rated fitness. The score represents the response to five questions on perceived overall fitness, CRF, muscular strength, speed-agility, and flexibility. Each question asks the participant to rate their own level of fitness as compared to people in the same age, and then rank each fitness type as either “very poor” (1), “poor” (2), “average” (3), “good” (4), or “very good” (5). The IFIS questionnaire has been translated to nine different languages (available here: http://www.helenastudy.com/ifis.php), of which the Swedish version, with minor modification in the text to fit an adult population, was used in the present study. The distribution of answers to all five IFIS questions is presented in the results section of this study. However, as no objective fitness test to assess speed-agility or flexibility was performed, the rest of the analyses included IFIS for *overall*, *CRF* and *strength* only.

### Objectively measured physical fitness

*CRF* was assessed using the 6-min walk test (6MWT), which is a feasible test to conduct during pregnancy^22,23,25,26^. The test was performed in a 30-m corridor where the women were instructed to walk back and forth as many times as possible during a 6-min period. The total distance walked (in m) was noted. As an estimate of exertion, heart rate was also measured during the test using the activity monitor watch Polar M400 (Polar Electro Oy, Kempele, Finland).

*Muscular strength* of the upper body was assessed using the handgrip strength test with an analogue dynamometer (TKK 5001, Grip-A, Takei, Tokyo, Japan). Prior to the test, the participant’s hand size was measured and the dynamometer was adjusted according to each individual grip span to enable maximum strength in the handle^27^. During the test, the women stood up and were instructed to hold their arm down beside their body. To ensure correct technique, the women were told to keep their arm alongside, but without support from (touching) their body. The women were then instructed to squeeze the dynamometer as hard as possible for a few seconds, two times with each hand. The best result from each hand (in kg) were then averaged and used in the analysis.

### Body composition

Body height was measured using standard procedures, and weight and body composition were measured through air-displacement plethysmography using the BodPod (COSMED) as previously described^22,23^. With this technique, body density can be calculated from the measured body volume and weight. Then, through the ‘two component model’ and by using reference densities, the body mass can be calculated and divided into fat-free mass (FFM) and fat mass (FM)^28,29^. Since the measurements for the present study were conducted in early pregnancy, calculations were adjusted using appropriate fat-free mass (1.098 g/cm^3^) and fat mass (0.900 g/cm^3^) densities for gestational week 14 to provide accurate estimates of body composition^23^. In addition to the standard measure of body size i.e., BMI, calculated as weight (kg) divided by height squared (m^2^), two body composition variables were used in the present study using measurement data from the BodPod: fat-free mass index (FFMI) and fat mass index (FMI). These index values were calculated as FFM (kg) or FM (kg) divided by height squared (m^2^), respectively.

### Cardiometabolic risk factors

The procedures for blood pressure measurement and blood sampling have been described previously^22,23^. In brief, a venous blood sample was drawn in the morning after an overnight fast. The blood sample was used to analyze metabolic parameters including fasting glucose levels, insulin, triglycerides, high-density lipoprotein (HDL) and total cholesterol. This was performed at the Department of Clinical Chemistry, Linköping University, Linköping, Sweden, which is accredited for these types of analyses (ISO/IEC 17025). Furthermore, insulin resistance was estimated using the homeostatic model assessment for insulin resistance (HOMA-IR), which was calculated as: (fasting insulin [mlE/L] × fasting glucose [mmol/L])/22.5^30^. Blood pressure was measured after a 5-min rest, where the average of two measurements (or three, if the first two differed more than 10 mmHg) were calculated for systolic or diastolic blood pressure, respectively. Mean arterial blood pressure (MAP) was calculated as: diastolic blood pressure + [0.333 × (systolic blood pressure − diastolic blood pressure)]^31^.

To assess whether self-reported and/or measured fitness is associated with cardiometabolic risk in early pregnancy, we calculated a composite cardiometabolic risk score. This risk score was composed of the sum of the standardized z-scores for FMI, triglycerides/HDL ratio, MAP, and HOMA-IR. This formula is based on the metabolic syndrome risk score previously used in an IFIS validation study in young adults^8^, and its construct validity has been demonstrated by Solera-Martinez et al. (2011) using confirmatory factor analysis^31^. However, due to our study population being pregnant, we used FMI instead of waist circumference in our calculation.

### Statistical analyses

All statistical analyses and plotting of data were performed in RStudio (version 1.3.959) using the programming language R (version 4.0.4; 2021-02-15), and the R packages *ggplot2* and *cowplot*. Before analyses, data were cleaned and checked for any missing values, and normality checks were performed on all relevant variables.

First, the validity of the IFIS in early pregnancy (*Aim 1*) was examined by comparing the average measured fitness level between the different categories of self-reported fitness. This was examined by one-way between-subjects analysis of variance (ANOVA), where measured fitness variables were entered as the dependent variable and their corresponding self-reported fitness variable entered as the independent variables (e.g., 6MWT ~ IFIS-CRF). The analyses presented are unadjusted since potential covariates such as age, parity, education level and country of birth, had no or only little relationship with the outcome, and consequently did not attenuate the results of the association between self-reported and measured fitness. To account for the self-reported IFIS question on overall fitness, we also computed a composite fitness score calculated as the average of the standardized z-scores ([value − mean]/standard deviation) from the two different objective fitness tests (handgrip and 6MWT), similarly to a previous study^9^. To determine which IFIS groups differed from each other, pairwise comparisons were performed using the Tukey Honest Significant Differences (HSD) post-hoc test for multiple comparisons of means. In addition, the relationship between self-reported and measured fitness for each component of physical fitness was also tested using a non-parametric interclass correlation of rankings (Spearman’s rho).

Next, the relationship of self-reported and measured fitness with body composition and cardiometabolic risk (*Aim 2*), were studied by using ANOVA. Since relatively few women scored their fitness level in the extreme categories, for these set of analyses the participants were merged to form three self-reported fitness levels i.e., “poor/very poor”; “average”; “good/very good”. For each body composition variable (BMI, FMI and FFMI) and cardiometabolic risk score, the difference in group means across fitness levels based on self-reported overall fitness, CRF and strength were assessed and compared with that of corresponding measured fitness variables (composed fitness score, 6MWT and handgrip rest, respectively). As three levels were used for self-reported fitness, participants were also classified into three levels for each measured fitness variable, based on the 25th and 75th percentiles: low (< P25), medium (P25–P75) and high (> P75). If statistically significant overall difference was evident across groups, ANOVA was followed by Tukey HSD post-hoc test for multiple comparisons of means between groups.

The measured fitness variables were normally distributed; however, due to skewed distribution of most body composition and cardiometabolic variables, those were log-transformed using the natural logarithm (ln) before used in further analyses (and before calculating the cardiometabolic risk score). Finally, for easier and more meaningful interpretation of results, all continuous variables were standardized using z-scores (where a z-score of 1 would be interpreted as 1 standard deviation above the mean). This enabled comparison between self-reported and measured fitness in relation to body composition and cardiometabolic risk variables by plotting them next to each other and with both overall fitness, CRF and strength categories included and compared in the same line plots. The level of statistical significance was set as p < 0.05 for all tests.

## Results

### Participants’ characteristics and physical fitness levels

**Figure 1:**
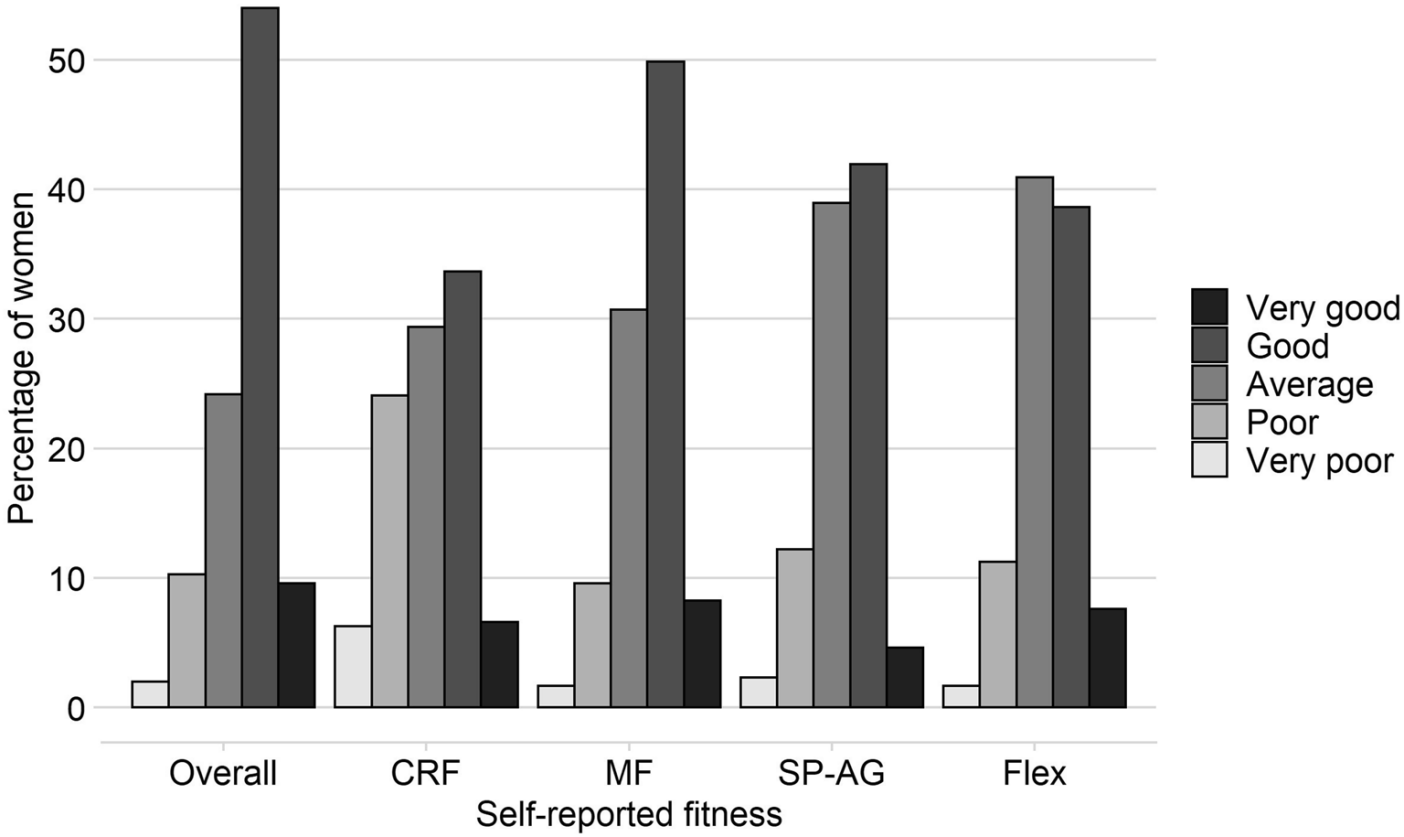
Distribution of answers to the five questions on physical fitness using the International FItness Scale (IFIS). Women in early pregnancy (n = 303, except for Overall Fitness where n = 302). *Overall* overall physical fitness, *CRF* cardiorespiratory fitness, *Strength* muscular strength, *SP-AG* speed-agility, *Flex* flexibility.

Table 1 provides information on the women’s background characteristics, body composition, cardiometabolic risk variables, and self-reported and measured fitness levels. In general, study participants were young women (mean age 31 years), highly educated (78% university degree), and most of them (88%) were born in Sweden. Figure 1 shows the distribution of answers to the IFIS questions on self-reported fitness. Most women classified their overall fitness, CRF, muscular strength and speed-agility as “good”, whereas most women classified their flexibility as “average”.

**Table 1:** Participants’ characteristics, cardiometabolic health variables and fitness levels of women pregnant in gestational week 14 (n = 303).

	Value^a^	Min–Max
**Characteristics**		
Age (years)	31 ± 4	20–44
Educational attainment (%)		
Primary school (9 years)	0.7 (2)	
High school (12 years)	21.4 (65)	
University degree	78.0 (236)	
Parity (%)		
0	57.8 (175)	
≥ 1	42.2 (128)	
Birth country (%)		
Sweden	88.4 (268)	
Other country	11.6 (35)	
Smoking before pregnancy^b^ (%)	2.0 (6)	
**Body composition**		
Weight (kg)	67.6 ± 11.6	44.7–120.1
Height (m)	1.67 ± 0.06	1.46–1.82
BMI (kg/m^2^)	24.2 ± 3.8	17.4–41.1
FMI (kg/m^2^)	7.9 ± 3.2	3.6–22.7
FFMI (kg/m^2^)	16.3 ± 1.3	12.8–20.0
**Cardiometabolic risk variables**		
Glucose^c^ (mmol/L)	4.8 ± 0.3	3.3–5.8
Insulin (mlE/L)	6.4 ± 3.0	1.7–19.0
HOMA-IR^c,d^	1.4 ± 0.7	0.4–4.5
Diastolic blood pressure (mmHg)	70 ± 6	54–96
Systolic blood pressure (mmHg)	108 ± 8	91–140
MAP^e^	83 ± 7	66–110
Total cholesterol (mmol/L)	4.6 ± 0.7	3.1–6.9
HDL cholesterol (mmol/L)	2.0 ± 0.3	1.1–3.0
Triglycerides (mmol/L)	1.0 ± 0.4	0.4–3.0
**Measured physical fitness**		
6-min walk test (m)	670 ± 55	497–803
Hand-grip strength test (kg)	33.2 ± 5.1	13.8–49.8
**Self-reported physical fitness**^f^		
Overall fitness^c^	3.6 ± 0.9	1–5
Cardiorespiratory fitness	3.1 ± 1.0	1–5
Muscular strength	3.5 ± 0.8	1–5
Speed-agility	3.3 ± 0.8	1–5
Flexibility	3.4 ± 0.8	1–5

*BMI* body mass index, *CRF* cardiorespiratory fitness, *FMI* fat mass index, *FFMI* fat-free mass index, *HDL* high density lipoprotein, *HOMA-IR* homeostatic model assessment for insulin resistance, *MAP* mean arterial blood pressure.

^a^Values shown are mean ± standard deviation for continuous variables, or % (n) for categorical variables.

^b^None of the women reported smoking during pregnancy (gestational week 14).

^c^n = 302 (one had missing value for fasting blood glucose and one for self-reported overall fitness).

^d^HOMA-IR was calculated as: (fasting insulin [mlE/L] × fasting glucose [mmol/L])/22.5.

^e^MAP was calculated as: diastolic blood pressure + [0.333 × (systolic blood pressure − diastolic blood pressure)].

^f^Assessed through questionnaires using the International FItness Scale (IFIS).

### Validity of self-reported (IFIS) against measured fitness in early pregnancy

**Figure 2:**
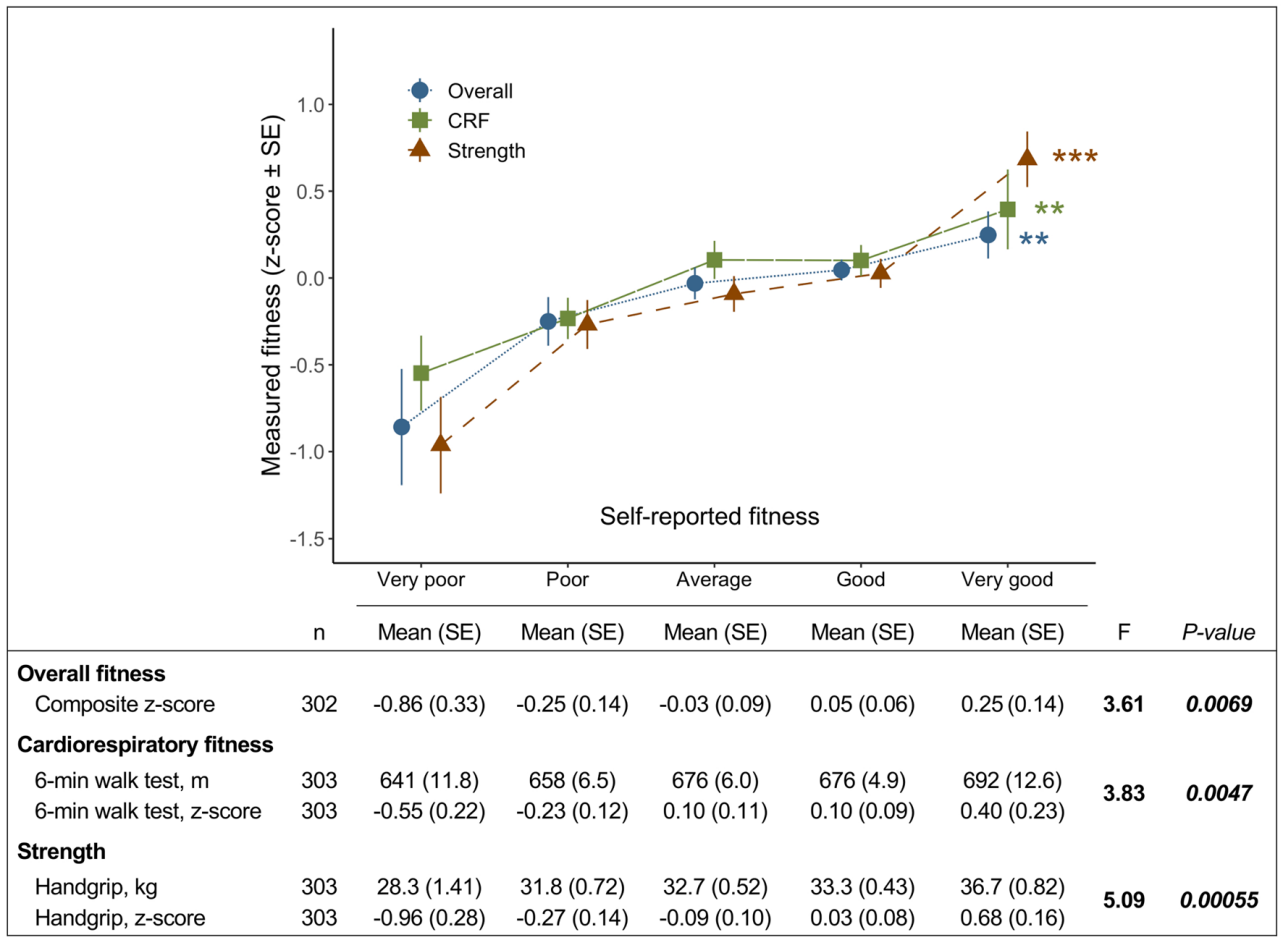
Comparison between self-reported (International FItness Scale, IFIS) and measured fitness in early pregnancy. The plot shows group means with standard error (SE) for each level of self-reported fitness and the corresponding fitness test. Values shown are actual fitness test results as well as standardized z-scores for easier comparison. Self-reported overall fitness is compared with a composite score computed as the average of the z-scores from the two different fitness tests, i.e., handgrip and 6-min walk test. *CRF* cardiorespiratory fitness: self-reported CRF compared with 6-min walk test. Self-reported muscular strength is compared with handgrip strength test. F-statistics and p-value from one-way analysis of variance (ANOVA). Significance level from the overall ANOVA test for each fitness type is indicated with symbols: ‘***’ p < 0.001, ‘**’ p < 0.01, ‘*’ p < 0.05.

Women who reported higher overall fitness, CRF or muscular strength also had higher scores on the corresponding measured fitness tests. As can be seen in Fig. 2, the relationship between self-reported and measured fitness appears to be linear, and there was a significant difference in measured fitness across the five self-reported levels for both overall fitness (overall p = 0.0069), CRF (overall p = 0.0047) and strength (overall p = 0.00055). Tukey’s pairwise comparison of group means showed statistically significant difference primarily between the lowest (“very poor”) and highest (“very good”) fitness groups for all fitness types assessed (Tukey HSD test p = 0.013 for overall fitness, p = 0.025 for CRF, and p = 0.0057 for strength). On group level, the difference in the performance of the 6MWT between those who reported “very poor” and “very high” CRF was on average 51 m, and 8.4 kg in the handgrip strength test (mean and standard error for each group is shown in Fig. 2). Furthermore, Spearman’s rank-order test confirmed a statistically significant, although weak, positive correlation between self-reported and measured fitness (overall fitness ρ = 0.16, p = 0.0045; CRF ρ = 0.17, p = 0.0023; strength/muscular fitness ρ = 0.23, p < 0.001).

### Physical fitness in relation to body composition and cardiometabolic risk

**Figure 3:**
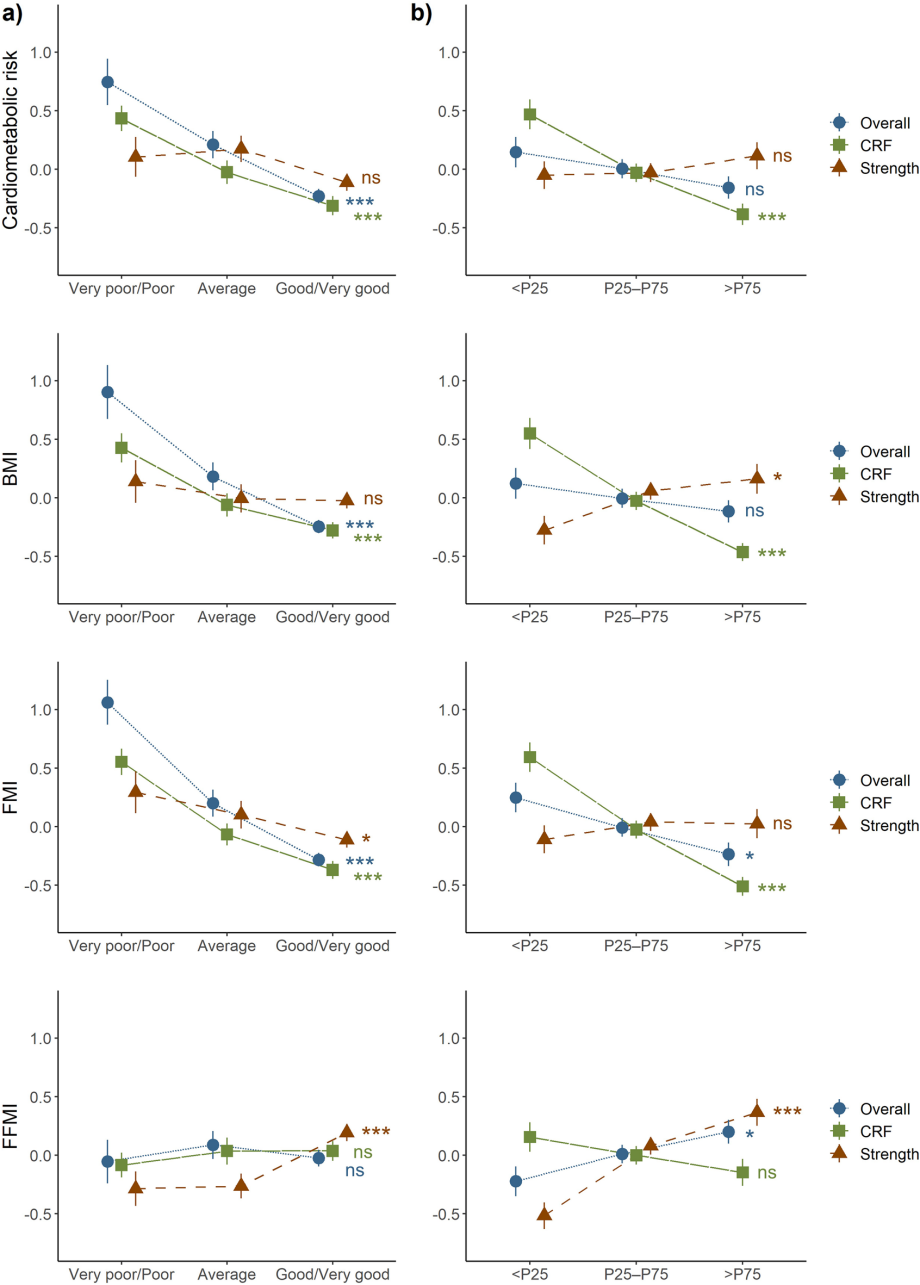
Relative differences in cardiometabolic risk score and body composition according to categories of (**a**) self-reported and (**b**) objectively measured physical fitness in early pregnancy. Measured fitness is split in percentiles indicating relative low (> P25), medium (P25-P75) and high (> P75) fitness levels. Mean and standard error bars using standardized z-scores (after log-transformation) are shown for each group and category. Overall fitness: self-reported overall fitness compared with a composite score computed as the average of the z-scores from the two different fitness tests, i.e., handgrip and 6-min walk test. CRF, cardiorespiratory fitness: self-reported CRF compared with 6-min walk test. Strength: self-reported muscular strength compared with handgrip test. *BMI* body mass index, *FMI* fat mass index, *FFMI* fat-free mass index. Significance level from the overall ANOVA test for each fitness type is indicated with symbols: ‘***’ p < 0.001, ‘**’ p < 0.01, ‘*’ p < 0.05, and ‘ns’, non-significant.

Considering the low number of individuals in the extreme self-reported fitness categories (i.e., those who classified their own fitness level as very low or very high), three fitness levels were used when investigating the relationship of self-reported and measured fitness with body composition and cardiometabolic risk (*Aim 2*). Figure 3 shows the association of self-reported fitness (panel a) and measured fitness (panel b) levels with cardiometabolic risk score and body composition measures, and corresponding results from the ANOVA and post-hoc pairwise comparison tests can be found as Supplementary Table S1 online. In general, the associations were consistent for self-reported and measured fitness, especially for CRF: higher level of CRF was related to lower cardiometabolic risk as both self-reported and measured CRF showed a negative association with the cardiometabolic risk score (overall p < 0.001 for both). Also, self-reported overall fitness showed the same association pattern. However, no association with the cardiometabolic risk score was observed with muscular strength (neither self-reported nor measured), and also not for the overall variable for measured fitness, which was a composed score combining both 6MWT and handgrip test results. Results from the individual components used to calculate the cardiometabolic risk score is presented in the Supplementary Table S1 and Fig. S1 online.

Next, as further shown in Fig. 3, both self-reported and measured CRF showed significant and similar association with BMI as well as FMI measured with the BodPod (overall p < 0.001 for both). Self-reported overall fitness also showed strong association with both of these body composition measures. The associations with strength varied somewhat between self-reported and measured fitness, although it showed no or little association with BMI and FMI. Finally, as expected FFMI showed association only for muscular strength (self-reported and measured; overall p < 0.001 for both) but not for CRF: women who reported higher muscular strength or performed better on the handgrip test also tended to have higher FFMI.

### Sensitivity analyses

To address the robustness of the findings we conducted some sensitivity analyses. First, considering the low number of individuals in the extreme self-reported fitness categories, we also tested the validity of the IFIS (*Aim 1*) using three fitness levels (“poor/very poor”; “average”; “good/very good”) instead of all five. The results and conclusions were similar showing an overall significant difference across groups for both overall fitness (overall p = 0.0093, F = 4.8), CRF (overall p = 0.0025, F = 6.1) and strength (overall p = 0.018, F = 4.1), and most between-group differences observed between the highest and lowest self-reported fitness groups. The trend remained approximately linear for overall fitness and strength, while the IFIS for CRF appeared to discriminate women with lower CRF levels from the rest, i.e. women reporting “poor/very poor” CRF had significantly lower measured CRF than those reporting their CRF as either “average”, “good” or “very good”.

Next, as individual motivation to perform in the 6MWT may influence test results and consequently our findings, we utilized the data on heart rate (HR) during the test, as an estimate of exertion. Average HR was available for 290 (96%) of the women, and in a second sensitivity analysis we assessed the validity of self-reported CRF with measured CRF by including only data from women (n = 247) with an average HR during the 6MWT of > 60% of their estimated maximum HR. Again, results were similar, and the conclusions remained the same. Furthermore, using the same subset of data in the association analyses with the cardiometabolic risk score (*Aim 2*), as expected the relationship between measured CRF and cardiometabolic risk became slightly stronger than with the whole sample, with an average difference in risk score of 1.1 SD between the lowest (< P25) and highest (> P75) measured CRF fitness groups.

Finally, in order to address whether the choice of splitting the fitness data in the percentiles as described above (< P25, P25-P75, > P75) had any impact on the results for aim 2, we also performed the same analysis using three tertiles (i.e., < P33.3, P33.3-P66.6, > P66.6). These results were very similar (see Supplementary Fig. S2 online) and therefore, conclusions remained the same.

## Discussion

Our findings support the usefulness of the IFIS in early pregnancy, as women in the study who reported higher physical fitness also had significantly better measured fitness levels on a group level compared to women reporting lower fitness. In addition, IFIS provides concordant associations with cardiometabolic risk factors compared to associations based on objectively measured physical fitness. Although not recommended to be used on an individual level yet, the results of this study indicate that the IFIS may offer a valuable tool for assessing fitness levels of pregnant women in large population-based studies where traditional measuring of fitness is not feasible.

First, we found a weak but statistically significant linear association between self-reported and objectively measured physical fitness in a way that higher self‐reported fitness, regardless of the fitness component, was related to better scores in the measured fitness tests. This is in concordance with the validity study of IFIS in young adults (72% women) where self-reported fitness showed agreement with measured fitness^8^. Our findings are also similar to what has been recently reported by Romero-Gallardo et al. (2020)^9^, who were the first (and only) to investigate the validity of IFIS in early pregnancy (gestational week 16) for a subpopulation of pregnant women (n = 106) included in the GESTAtion and FITness (GESTAFIT) project in Spain. A key finding in that study was that the IFIS seemed to be able to discriminate women with lower CRF levels (“poor/very poor”) from those with “average” or “good/very good” CRF levels. Notably, using the same approach (see sensitivity analysis above) we observed the same phenomenon for CRF also in the present study, even though different tests for measuring CRF were used (6MWT in the HealthyMoms population and Bruce test in GESTAFIT). Furthermore, the validation results for strength were also comparable across the two studies, observing agreement between self-reported strength and handgrip test results^9^. Thus, our study confirms the previous findings by demonstrating usefulness of the IFIS also in another population using a larger sample size.

Measured CRF has previously been negatively associated with cardiometabolic risk factors in both pregnant^23^ and non-pregnant populations^1,2,8^. Also, the IFIS has been used in a few studies for assessing self-reported physical fitness and investigating its association to pregnancy-related outcomes, such as health-related quality of life^9^, less pain and pain-related disability during pregnancy^5^ as well as poor sleep and tiredness-fatigue^21^. However, to the best of our knowledge, no previous study has investigated and compared the association with self-reported versus measured fitness with cardiometabolic health in pregnancy. Thus, this was the second aim of our study, and interestingly the IFIS seemed to perform similarly well as measured fitness in this population. Hence, the second key finding in our study is the consistency of the relationship between self-reported and measured fitness, especially CRF, with cardiometabolic risk factors and body composition in early pregnancy. More specifically, the results showed a linear relationship indicating a lower cardiometabolic risk for women who reported themselves as more “fit” or performed better in the 6MWT. This is in line with previous findings by Ortega and colleagues, who also investigated the relation between fitness levels (IFIS vs measured) and the corresponding cardiometabolic risk score in young adults, and found especially higher overall self-reported fitness (IFIS) and measured CRF to be associated with lower cardiometabolic risk score^8^. Moreover, in our study we observed a significant association between higher muscular strength (both self-reported and measured) and higher FFMI. This is expected since a higher FFMI indicates a higher muscle mass. Finally, in our study not only measured fitness but also self-reported fitness showed similar association with both FMI and BMI. This is interesting as FMI, when measured using state-of-the-art methodology (i.e., air-displacement plethysmography in a BodPod), is generally considered a more accurate measure of body composition than BMI.

In addition to providing further evidence on the usefulness of the IFIS in early pregnancy, this is the first study to investigate the level of consistency of the relationship of self-reported versus objectively measured physical fitness with cardiometabolic risk factors in pregnant women. The strengths of the present study include a relatively large sample of pregnant women, with carefully collected information on both self-reported and objectively measured fitness as well as cardiometabolic risk factors for more than 300 women. Also, using accurate body composition methodology to derive FM and FFM allowed us to compare associations with BMI against corresponding associations with FMI^28^. Moreover, clustering of individual cardiometabolic risk factors has been reported to associate stronger with adverse pregnancy outcomes than a single risk factor^32^. In this study we used a composite score to assess cardiometabolic risk, with a formula based on the risk score previously used in the IFIS validation study in young adults^8^, and for which construct validity has been demonstrated earlier^31^. The use of the IFIS provides a valuable option to clinical practice in large-scale studies that is easy and quick to conduct, and all pregnant women can answer regardless of musculoskeletal disorders or any pregnancy-related adverse consequences that may hinder them in participating in a fitness test.

The study also has some limitations that need to be considered. Firstly, we were not able to include speed-agility or flexibility in the comparisons between self-reported and measured fitness as no such tests were performed in the HealthyMoms trial. Nevertheless, CRF and muscular strength are the two physical fitness components most consistently linked to health outcomes^1–3^. Secondly, in the present cross-sectional study we investigated the validity of the IFIS according to physical fitness levels in early pregnancy, but did not investigate test–retest reliability of the tool over time. However, a previous systematic review and meta-analysis of seven studies concluded that there were moderate to substantial reliability of the IFIS, although heterogeneity was observed among the studies^33^. Moreover, as is commonly the case in research studies, participants enrolled in our study had generally a higher education level (78% had a university degree) compared to the general population (40–45% of women in Sweden)^34^ and Swedish pregnant women in their first trimester (54.5%, based on data on 456,045 pregnancies in the Swedish Pregnancy Register between 2010 and 2018)^35^. However, age (31 ± 4 years) and BMI (24.2 ± 3.8 kg/m^2^) of the women in our study were still comparable to corresponding numbers of Swedish pregnant women in general (average age 30.7 ± 5.0 years and BMI 24.8 ± 4.7 kg/m^2^)^35^. Furthermore, our women reported slightly higher reported fitness scores when compared to pregnant Spanish women^9,21^, and performed slightly better in terms of the handgrip test and 6MWT as compared to other studies^4,9,36,37^. Nevertheless, we were still able to observe significant differences across fitness groups, and concordance between self-reported and measured fitness; however, it would be interesting to further validate the IFIS in a more heterogeneous group of pregnant women, including women with both very low and very high physical fitness. Finally, since the results are derived from a cross-sectional data sample, we cannot draw any conclusions about causality between physical fitness and cardiometabolic risk factors.

In conclusion, our findings suggest that self-reported fitness assessed with the IFIS could be a useful tool to stratify participants into physical fitness levels in large population-based pregnancy studies where objective fitness tests are not feasible or appropriate to conduct. The study adds to the literature as it demonstrates the usefulness of the IFIS in early pregnancy and also show statistically significant association with cardiometabolic risk and body composition measures. There is a need to cross-validate the IFIS in different study populations and ethnic groups before it can be used on a large-scale, and the present study contributes an important step in this process. From a public health perspective, the agreement of both self-reported and measured fitness with cardiometabolic risk factors are relevant since an easy-to-administer questionnaire may be useful within maternity care to obtain information on fitness levels as a compliment tool when assessing women’s health status. However, future studies are needed to understand whether self-reported fitness can be used in the clinical setting on an individual level, and furthermore, should also investigate the ability of the IFIS to predict long-term cardiometabolic health outcomes.

## Supplementary Information

Supplementary Information.

## Data Availability

The datasets analyzed during the current study are not publicly available due to restrictions in the ethical approval according to our national ethical guidelines, but are available from the corresponding author on reasonable request.
