# Supplementary material for: Self-reported (IFIS) versus measured physical fitness, and their associations to cardiometabolic risk factors in early pregnancy
Source: Sci Rep. 2021 Nov 22;11:22719. doi: 10.1038/s41598-021-02149-7 (PMC8608964; doi:10.1038/s41598-021-02149-7)
Supplement: Supplementary file 1 — Supplementary Information. [file 41598_2021_2149_MOESM1_ESM.pdf]

Supplementary information

Self-reported (IFIS) versus measured physical fitness, and their associations to cardiometabolic risk factors in early pregnancy

Maria Henström[1,*], Marja H Leppänen[1,3,4], Pontus Henriksson[2], Emmie Söderström[2], Johanna Sandborg[1,2], Francisco B Ortega[1,5], and Marie Löf[1,2]

[1] Department of Biosciences and Nutrition, Karolinska Institutet, 141 83 Huddinge, Sweden
[2] Department of Health, Medicine and Caring Sciences, Division of Society and Health, Linköping University, 581 83 Linköping, Sweden
[3] Folkhälsan Research Center, Helsinki, Finland
[4] Faculty of Medicine, University of Helsinki, Helsinki, Finland
[5] PROFITH (PROmoting FITness and Health through physical activity) research group, Department of Physical Education and Sports, Faculty of Sport Sciences, Research Institute of Sport and Health, University of Granada, Spain
* email: maria.henstrom@ki.se

List of supplementary files:

Supplementary Table S1. Self-reported and measured fitness levels association with cardiometabolic risk factors and body composition in early pregnancy.

Supplementary Figure S1. Relative differences in the cardiometabolic health variables included in the cardiometabolic risk score according to levels of a) self-reported and b) objectively measured physical fitness in early pregnancy. Measured fitness is split in percentiles indicating relative low (>P25), medium (P25-P75) and high (>P75) fitness levels.

Supplementary Figure S2. Relative differences in cardiometabolic risk score and body composition according to categories of a) self-reported and b) objectively measured physical fitness in early pregnancy. Measured fitness is split in tertiles (approx. 33% of women in each).

Supplementary Table S1. Self-reported and measured fitness levels association with cardiometabolic risk factors and body composition in early pregnancy.

			Self-reported fitness (IFIS)[1]						Measured fitness[2]					
			ANOVA[3]		Pairwise comparisons[4]				ANOVA[3]		Pairwise comparisons[4]			
	Fitness	n	F-value	*P-value*	1—2	2—3	1—3	n	F-value	*P-value*	1—2	2—3	1—3
**Cardiometabolic risk[5]**													
Risk score	Overall	301	18.93	***<0.001****	*	**	***	302	1.76	*ns*			
	CRF	302	16.04	***<0.001****	**	*ns*	***	302	14.29	***<0.001****	***	*	***
	Strength	302	2.72	*ns*				302	0.63	*ns*			
**Body composition**													
Body mass index (BMI)	Overall	302	25.61	***<0.001****	***	**	***	303	1.09	*ns*			
	CRF	303	14.43	***<0.001****	**	*ns*	***	303	21.03	***<0.001****	***	**	***
	Strength	303	0.38	*ns*				303	4.14	***<0.05****	*	*ns*	*
Fat mass index (FMI)	Overall	302	37.02	***<0.001****	***	***	***	303	4.56	***<0.05****	*ns*	*ns*	**
	CRF	303	26.45	***<0.001****	***	*ns*	***	303	25.54	***<0.001****	***	***	***
	Strength	303	3.06	***<0.05****	*ns*	*ns*	*ns*	303	0.58	*ns*			
Fat-free mass index (FFMI)	Overall	302	0.39	*ns*				303	3.49	***<0.05****	*ns*	*ns*	*
	CRF	303	0.47	*ns*				303	1.65	*ns*			
	Strength	303	8.30	***<0.001****	*ns*	***	*	303	16.89	***<0.001****	***	*ns*	***
**Cardiometabolic health**													
HOMA-IR	Overall	301	9.28	***<0.001****	*ns*	**	**	302	1.17	*ns*			
	CRF	302	11.35	***<0.001****	*ns*	*	***	302	6.48	***<0.01****	*ns*	*	**
	Strength	302	1.11	*ns*				302	1.13	*ns*			
MAP	Overall	302	0.35	*ns*				303	0.07	*ns*			
	CRF	303	0.86	*ns*				303	1.71	*ns*			
	Strength	303	0.96	*ns*				303	0.25	*ns*			
Triglycerides	Overall	302	6.95	***<0.01****	*ns*	*ns*	***	303	0.76	*ns*			
	CRF	303	4.81	***<0.01****	*ns*	*ns*	**	303	5.05	***<0.01****	*	*ns*	**
	Strength	303	1.63	*ns*				303	1.14	*ns*			
HDL cholesterol	Overall	302	5.44	***<0.01****	*ns*	*ns*	*	302	1.82	*ns*			
	CRF	303	6.16	***<0.01****	*ns*	*ns*	**	302	1.61	*ns*			
	Strength	303	3.26	***<0.05****	*ns*	*ns*	*ns*	302	1.67	*ns*			

[1] Self-reported overall fitness, CRF and strength, assessed through questionnaires using the International FItness Scale (IFIS).
[2] Measured CRF: 6-minute walk test. Measured strength: handgrip test. A composite overall fitness score was computed as the average of the standardized (z) scores ([value - mean]/standard deviation) from the two different objective fitness tests.
[3] One-way analysis of variance analysis (ANOVA).
[4] Pairwise comparisons using the Tukey's honest significance test for multiple comparisons of means. Fitness levels compared: 1 = low fitness level, defined as "Very Poor/Poor" (IFIS) or measured fitness <P25; 2 = average fitness level, defined as "Average" (IFIS) or measured fitness P25–P75; 3 = high fitness level, defined as "Good/Very good" (IFIS) or measured fitness >P75. Significance level symbols: '***' p<0.001, '**' p<0.01, '*' p<0.05, and 'ns', non-significant.
[5] Cardiometabolic risk score was computed as the sum of standardized z-scores for FMI, triglycerides/HDL ratio, MAP, and HOMA-IR. MAP was calculated as: diastolic blood pressure + [0.333 x (systolic blood pressure - diastolic blood pressure)]. HOMA-IR was calculated as: (fasting insulin [mIE/L] × fasting glucose [mmol/L])/22.5.
Skewed variables were log-transformed before standardized z-scores were computed and used in the analyses.
Abbreviations: BMI, body mass index; CRF, cardiorespiratory fitness; FMI, fat mass index; FFMI, fat-free mass index; HDL, high density lipoprotein; HOMA-IR, homeostatic model assessment for insulin resistance; MAP, mean arterial blood pressure; ns, non-significant; SE, standard error.

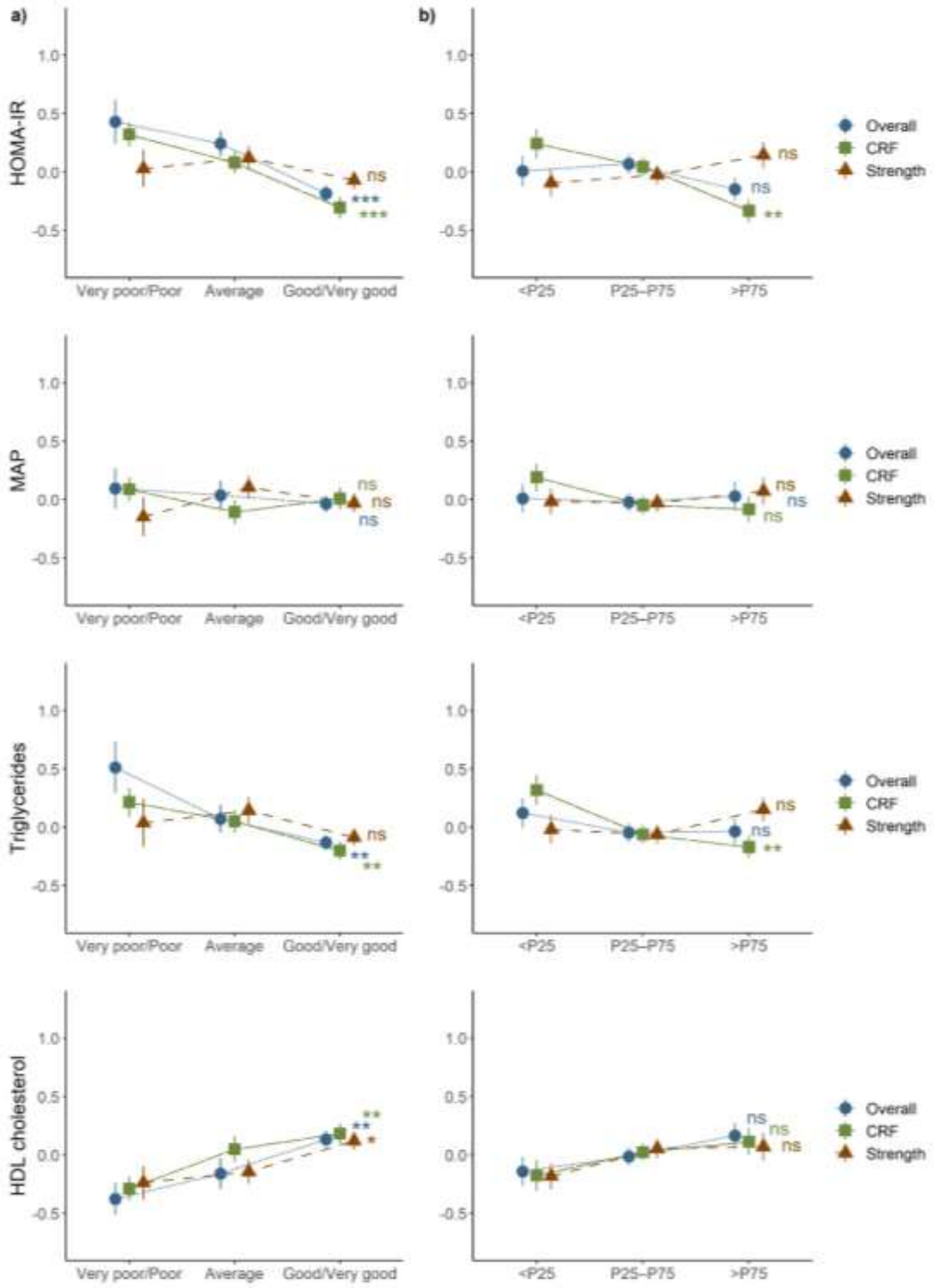

Supplementary Figure S1. Relative differences in the cardiometabolic health variables included in the cardiometabolic risk score according to levels of a) self-reported and b) objectively measured physical fitness in early pregnancy. Measured fitness is split in percentiles indicating relative low (>P25), medium (P25-P75) and high (>P75) fitness levels. Mean and standard error bars using standardized z-scores (after log-transformation) are shown for each group and category. MAP was calculated as: diastolic blood pressure + [0.333 x (systolic blood pressure - diastolic blood pressure)]. HOMA-IR was calculated as: (fasting insulin [mIE/L] × fasting glucose [mmol/L])/22.5.

Abbreviations: HDL, high density lipoprotein; HOMA-IR, homeostatic model assessment for insulin resistance; MAP, mean arterial blood pressure.

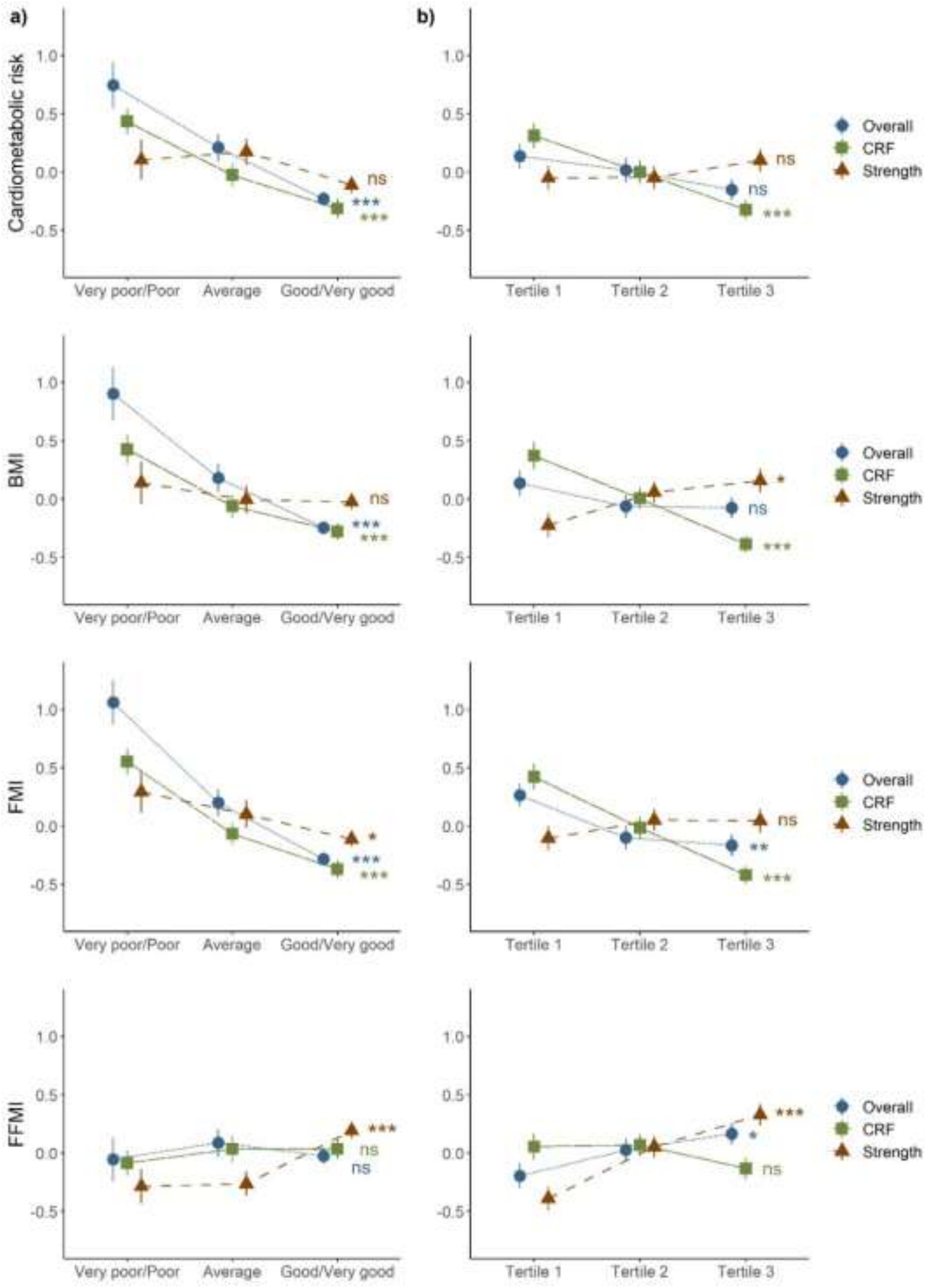

Supplementary Figure S2. Relative differences in cardiometabolic risk score and body composition according to categories of a) self-reported and b) objectively measured physical fitness in early pregnancy. Measured fitness is split in tertiles (approx. 33% of women in each). Mean and standard error bars using standardized z-scores (after log-transformation) are shown for each group and category. Overall fitness: self-reported overall fitness compared with a composite score computed as the average of the z-scores from the two different fitness tests, i.e., handgrip and 6-min walk test. CRF, cardiorespiratory fitness: self-reported CRF compared with 6-min walk test. Strength: self-reported muscular strength compared with handgrip test. BMI, body mass index; FMI, fat mass index; FFMI, fat-free mass index. Significance level from the overall ANOVA test for each fitness type is indicated with symbols: '***' p<0.001, '**' p<0.01, '*' p<0.05, and 'ns', non-significant.
